# The value of blue-green algae (*Spirulina platensis)* as a nutritive supplement and toxicant against almond moth [*Cadra cautella* (Lepidoptera: Pyralidae)]

**DOI:** 10.1371/journal.pone.0259115

**Published:** 2021-10-26

**Authors:** Wahidah H. Al-Qahtani

**Affiliations:** Department of Food Sciences and Nutrition, College of Food and Agriculture Sciences, King Saud University, Riyadh, Saudi Arabia; Ghazi University, PAKISTAN

## Abstract

Blue-green algae, *Spirulina platensis* is a well-known algal formulation known for its beneficial effects on the growth and development in several types of organisms. Although it is used as a food supplement, it possesses significant toxic effects on growth and development of organisms. This study assessed the positive/negative impacts of *S*. *platensis* on almond moth, *Cadra cautella* (almond moth) that is a serious pest of date fruits and other grains under laboratory conditions. The *S*. *platensis* powder were mixed with diet and newly hatched *C*. *cautella* larvae were fed. The larvae were observed on alternate days to record the data. The diet was changed once a week. The *S*. *platensis* proved very good nutrition supplement at lower dose. Whereas, moderate and high mortality was noted for 5 and 10% formulations, respectively. Moreover, larval span was significantly altered by different formulations and lower formulation (1%) resulted in shorter larval period compared to the rest of the formulations. Although 33% mortality was recorded under 5% *S*. *platensis* formulation, however, the larvae which reached to adult stage, copulated, and females laid more eggs. Furthermore, the highest mortality (90%) was observed under 10% *S*. *platensis* formulation and a few larvae reached adult stage; thus, no data on pupal period and reproductive traits was recorded for this formulation. These findings proved that *S*. *platensis* can be used as nutritional supplement as well as a toxic substance to manage *C*. *cautella* in date storage. However, future studies on this are needed to reach concrete conclusions.

## Introduction

Date palm, (*Phoenix dactylifera* L.) is of high economic importance and grown in many countries of the world, including Saudi Arabia [1]. Many crops under field conditions and stored agricultural commodities, including dates, wheat grains, and legumes are attacked by almond moth, [*Cadra cautella* (Walker) (Lepidoptera: Pyralidae)]. It is a cosmopolitan insect having a wide host range, and damage caused by the pest reached to the highest level if timely control is not opted [2–4]. It is spread over several countries, including Saudi Arabia [5, 6].

Several studies have reported/determined the biology of almond moth using different media such as poultry-based diet [7], wheat flour, and dates [4]. The nutritive value of date fruit has been exclusively studied [8]. Juvenile stage of almond moth attacks date fruits. Larval stage of almond moth is very dangerous for the date fruits as they consume entire fruit and deteriorate aesthetic and economic value of the fruits. The current study assessed life history traits of almond moth on *Spirulina platensis* as dietetic supplement. Inferring the toxic effect of *S*. *platensis* on almond moth was the second major objective of the study.

Generally, all animals require some basic nutrients for their growth which come from their daily feed/food. The success of a species depends on its fertile progeny and proper transfer of the traits to the next generation. If neonates of the insects are not fed properly, they will not perform well in the future posing a serious threat for the extinction of the species. Therefore, developing stages must be fed properly to avoid this serious issue. *Spirulina platensis* is a well-known algal formulation known for its beneficial effects on the growth and development in several types of organisms [9, 10] as it provides complete nutrition for the proper growth and development.

Several recent studies have investigated the benefits of *S*. *platensis* in a variety of categories, ranging from nutritional advantages to hazardous consequences. Unfortunately, little investigation has been done on its anti-insect capabilities. It has been used in humans to treat micronutrient deficiencies in new-born kids as well as improving immune system. Different *S*. *platensis* based infant foods have been produced, and foods containing 5% *S*. *platensis* are found suitable for 1–3 years children. In addition, patients who used *S*. *platensis* supplements had higher hemoglobin levels. *Spirulina platensis* is known as super food and used for various purposes [11–15]. It is a nutrient-dense food that contains >100 nutrients and 62% protein, whereas protein percentage up to 77% has been recorded [11, 16]. *Spirulina platensis* has been studied in chickens to combat heat shock proteins [17].

The effect of *S*. *platensis* on insects has been studied in numerous ways. It was utilized as a protein and carbohydrate source in phytophagous ladybug beetle, *Henosepilachna vigintioctopunctata* (F.) (Coleoptera: Coccinellidae) [18]. The cotton leafworm, *Spodoptera littoralis* (Boisd.) (Lepidoptera: Noctuidae) was used to test the toxicity of *Spirulina*. *Spirulina platensis* proved harmful to larvae and other biological parameters and caused 100% mortality in 2^nd^ and 4^th^ instar larvae with 5% or above formulations [19, 20].

The goals of this study were to examine the effects of *S*. *platensis* either as a nutritive supplement or as a toxin on life cycle parameters of almond moth. Determining the impact of *S*. *platensis* on growth and development of almond moth larvae was the prime objective of the study. It was hypothesized that lower amount of *S*. *platensis* will serve as a nutritive substance, while higher doses will prove toxic for almond moth.

## Materials and methods

### Ethics statement

The adults of almond moth were directly collected from the date palm orchard situated in Riyadh region, Saudi Arabia (24.4164°N, 46.5765°E). We declare that almond moth was not collected from the public parks or protected areas. Moreover, it is not an endangered species. Hence, no permits were required to conduct the study.

### *Cadra cautella* colony

The *C*. *cautella* colony was maintained on an artificial diet primarily containing poultry feed ingredients, which have been used in several studies [2, 3]. Fully grown larvae were removed from the main rearing box, separated into males and females, kept in 50 g plastic cups, and provided with small amount of artificial diet. The larvae pupated and paired upon adult eclosion, allowed to copulate, and lay eggs.

### Eggs collection

**Fig 1:**
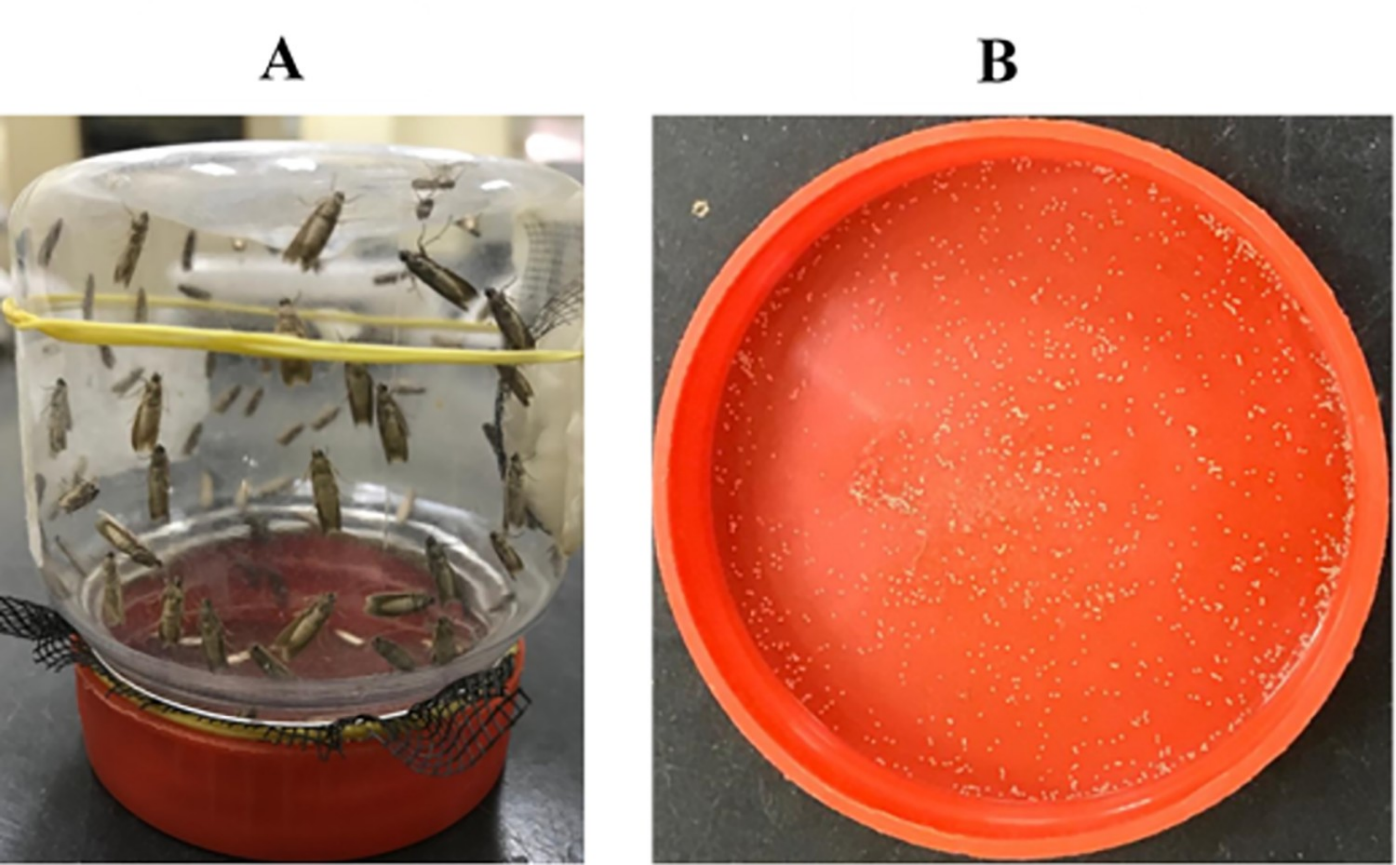
Mating arena and egg laying container for *Cadra cautella* adult moths (A) and cover containing *C*. *cautella* eggs (B).

The adult moths were paired in a 250 ml plastic jar. In total, 10–15 pairs were placed in the specified mating arena and egg laying container. The plastic jar, serving as a mating container, was cut from the lateral side to provide a 10% sugar solution. A piece of cotton was dipped in the sugar solution, covered with a piece of mesh, and placed on side of the jar within the cutting area. A rubber band was subsequently wrapped around the cotton to hold it securely. Opening of the jar was also supplied with mesh and lid was fixed above the mesh. The jar containing moth pairs was inverted. Female moths laid eggs that passed through the mesh and deposited in the jar cover (Fig 1). The eggs were cleaned from the jar cover by removing moth’s scales and used in the experiment.

### Preparation of *Spirulina*-supplemented diet

Five grams of artificial diet and required amount of *S*. *platensis* for each treatment were measured and weighed on an electric weighing apparatus (PGL 3002 Adam Equipment UK). The *S*. *platensis* was purchased from (Pharma CareEurope Ltd., UK) in powder form. Required amount of diet and *S*. *platensis* powder were thoroughly mixed and given to neonates. The diet and *S*. *platensis* mixing was done in a new, neat, and clean plastic cup to avoid any contamination. The diet was prepared only once and kept at 4°C in the refrigerator for further use. Before changing the larval diet, it was taken out of the refrigerator and kept at room temperature for at least one hour. The diet was provided to the larvae once temperature returned to normal.

### Eggs hatching and larvae used in bioassay

One-day old almond moth eggs were observed under microscope to remove broken and small sized eggs. The selected eggs (in a group of 50) were placed in the plastic cup and allowed to hatch at 25 ± 2°C and 60 ± 5% relative humidity (RH).

### Bioassay

Upon hatching, neonates were used for bioassay. There were five treatments, i.e., 0, 1, 2, 5, and 10% *S*. *platensis* mixed with artificial diet. The diet containing 0% *S*. *platensis* was considered as control treatment. Each treatment had three replications, and each replication contained ten larvae. Ten larvae were placed separately in a 50 g plastic cup and 0.25 g diet was supplied according to every treatment. All the larvae were placed in the incubator at 25 ± 2°C and 60 ± 5% RH after bioassay for further growth and development.

### Data collection

Larval growth was observed on alternate days to record data. All larvae were removed from the diet every week, dead larvae were counted and removed. The alive larvae were supplied with 0.25g fresh diet mixed with *S*. *platensis* according to treatments. The larvae were observed until all of them pupated in all treatments and larval duration was recorded. Larvae that succeed to pupate were observed daily and pupal period was noted. Upon eclosion of adults, moths from the pupae, male and female moths from the same treatment were paired (4 pair from each treatment) and the biological characteristic such as pre-oviposition, oviposition, post oviposition period, fecundity, hatchability, and life span were studied.

### Statistical analysis

The data were tabulated, compiled, and analyzed using general linear models procedure and one-way analysis of variance (ANOVA) (at p = 0.05), as statistical method with unbalanced number of samples (N) through SAS 9.2 [21]. Normality in the data was tested prior to ANOVA. The data were normally distributed; hence, analysis was performed on original, non-transformed data.

## Results

### Larval period

The blue-green algae, *Spirulina plateniensis* mixed with larval diet had significant impact on larval growth and development. The larval period was significantly different among treatments and larval growth was very slow with 10% formulation (*F =* 30.29; df = 4, 98; *P* < .0001). The shortest larval period was recorded for 1% formulation, which was less than the control treatment. Whereas, the longest larval period was observed with 10% formulation, which was significantly longer than the larval period in the control treatment (Table 1).

**Table 1:** Mean larval period (days ± SE) of *Cadra cautella* larvae reared on *Spirulina platensis* added artificial diet under laboratory conditions at 25 ± 2°C and 65 ± 5% relative humidity.

Treatments	Larval period
0% *Spirulina*	24.00 ± 0.28b
1% *Spirulina*	22.00 ± 0.26c
2% *Spirulina*	23.00 ± 0.35b
5% *Spirulina*	24.00 ± 0.22b
10% *Spirulina*	30.00 ± 1.00a

Means followed by the same letters do not differ significantly (*P* < 0.05).

### Larval mortality

Larval mortality was linearly related to *S*. *platensis* formulation percentage in the diet (Table 2). Initially, the larvae properly fed; however, feeding activity decreased/ceased with time, and larvae became week while feeding with higher *S*. *platensis* formulations. The larvae started to die within 1^st^ week under high *S*. *platensis* formulation. Most of the larvae under 10% formulation died within 2 weeks. The highest larval mortality was recorded for 10% treatment (Table 2). The overall mortality was significant among the treatments (*F =* 70.14; df = 4, 14; *P* < .0001).

**Table 2:** Mean larval mortality (% ± SE) of *Cadra cautella* larvae reared on *Spirulina platensis* added artificial diet under laboratory conditions at 25 ± 2°C and 65 ± 5% relative humidity.

Treatments	Larval mortality
0% *Spirulina*	13.33 ± 3.33c
1% *Spirulina*	6.66 ± 3.33c
2% *Spirulina*	26.66 ± 3.33b
5% *Spirulina*	33.33 ± 3.33b
10% *Spirulina*	90.00 ± 5.77a

Means followed by the same letters do not differ significantly (*P* < 0.05).

### Pupal period

Pupal span was recorded for the larvae that pupated successfully and adult moth eclosion was noted. The time between the day of pupation to adult eclosion was regarded as pupal period. Different *S*. *platensis* formulations significantly altered pupal period (*F =* 13.38; df = 4,98; *P* < .0001). The pupal period was two days longer under 1% *S*. *platensis* formulation compared to the rest of the treatments (Table 3).

**Table 3:** Mean pupal period (days ± SE) of *Cadra cautella* pupae reared on *Spirulina platensis* added artificial diet under laboratory conditions at 25 ± 2°C and 65 ± 5% relative humidity.

Treatments	Pupal period
0% *Spirulina*	8.84 ± 0.16 b
1% *Spirulina*	10.89 ± 0.25 a
2% *Spirulina*	9.68 ± 0.23 b
5% *Spirulina*	9.45 ± 0.18 b
10% *Spirulina*	9.00 ± 0.57 b

Means followed by the same letters do not differ significantly (*P* < 0.05).

### Reproductive traits

Pairing was done with 1:1 sex ratio after eclosion of adult male and female moths. Similar aged male and female moths were placed in a 50 g plastic cup and reproductive traits were observed. Female moths get ready for copulation after eclosion and start releasing pheromones within 6-12h. The male is attracted toward female and starts mating. Mating might last for few hours. Female lays eggs within 12–18 hours of mating. Time period before egg laying is considered as pre-ovipositional period. The pre-ovipositional period does not exceed 2–3 days in all the treatments and was not significant (*F =* 0.57; df *=* 3,15; *P*: 0.6445) (Table 4). The interval from egg laying till stop of egg laying is regarded as ovipositional period and it was not significant among different treatments (*F =* 0.44; df *=* 3, 15; *P*: 0.7256). Time period when female moth stop egg laying till death is known as post-ovipositional period. The post-ovipositional period (*F =* 1.00; df = 3, 15; *P =* 0.4262) and hatchability (*F =* 0.45; df = 3,15; *P =* 0.7208) were not altered by different *S*. *platensis* formulations, while fecundity was significantly affected and the female moth which were fed on 5% *S*. *platensis* formulation during the larval period laid more eggs as compare to the female moths in the control treatments (*F =* 7.75; *df =* 3, 15; *P =* 0.0038) (Table 4).

**Table 4:** Mean ovipositional periods (days ± SE), fecundity (numbers ± SE) and hatchability (% ± SE) of *Cadra cautella* reared on *Spirulina platensis* added artificial diet under laboratory conditions at 25 ± 2°C and 65 ± 5% relative humidity.

Treatments	Pre-ovipositional period	Ovipositional period	Post-ovipositional period	Fecundity	Hatchability
0% *Spirulina*	2.25 ± 0.25 a	5.5 ± 0.64 a	2.50 ± 0.50 a	193 ± 5.86 b	86 ± 1.75 a
1% *Spirulina*	2.5 ± 0.28 a	5.5 ± 0.64 a	1.50 ± 0.28 a	203 ± 6.01 b	87 ± 2.03 a
2% *Spirulina*	2.5 ± 0.28 a	5.0 ± 0.40 a	1.75 ± 0.25 a	211 ± 21.81 b	58 ± 1.86 a
5% *Spirulina*	2.75 ± 0.25 a	6.0 ± 0.70a	2.00 ± 0.57 a	272 ± 9.80 a	88 ± 2.10 a

Means followed by the same letters within each column do not differ significantly (*P* < 0.05).

### Adult life span

The interval from the day of the eclosion of adult moth till death was taken as adult life span. Adult life span was not significantly differed among different *S*. *platensis* formulations, except 5%. The female (*F =* 5.53; df *=* 3, 15; *P =* 0.0128) and male (*F =* 2.89; *df =* 3,15; *P =* 0.0793) adult moths, both survived longer under 5% *S*. *platensis* formulation compared to the control treatment (Table 5).

**Table 5:** Mean adult life span (days ± SE) of *Cadra cautella* pupae reared on *Spirulina platensis* added artificial diet under laboratory conditions at 25 ± 2°C and 65 ± 5% relative humidity.

Treatments	Female adult life span	Male adult life span
0% *Spirulina*	9.00 ± 0.00b	6.75 ± 0.62b
1% *Spirulina*	8.75 ± 0.25b	7.75 ± 0.25ab
2% *Spirulina*	8.50 ± 0.50b	7.25 ± 0.47ab
5% *Spirulina*	10.00 ± 0.00a	8.50 ± 0.28a

Means followed by the same letters within each column do not differ significantly (P < 0.05).

## Discussion

Exploring the effects of blue-green algae, *Spirulina platensis* on the phenological characteristics of almond moth demonstrated the importance of *S*. *platensis* against the pest. The findings suggested that *S*. *platensis* improved biological characteristics of almond moth at lower doses; however, higher doses caused higher mortalities and only a few larvae survived. Different formulations of *S*. *platensis* in diet significantly altered larval development duration and other life characteristics of almond moth. Previous studies have reported different developmental time for almond moth reared on various food medium [7, 22, 23]. Researchers have tried to find out the solution to manage almond moth by evaluating the toxic effects of carbon dioxide and ozone gases [24–26]. Molecular studies comprising transcriptome analysis to explore the reproduction control genes vitellogenin and its receptor gene and their silencing [27–29] and use of fungus [30] have been explored. However, all these tactics have certain limitations associated with them. The present study is comparatively economical, easy to apply and eco-friendly. The results of current studies are consistent with previous findings, where similar outcomes of *S*. *platensis* toxic properties against red palm weevil, *Rhynchophorus ferrugineus* (Olivier) (Coleoptera: Dryopthoridae) have been reported [31].

The *S*. *platensis* has several advantages and currently being used as a source of nutrition for birds, poultry, fish, and other animals [10, 32, 33]. There are few studies that have looked at its value as a dietary supplement and its insect-killing properties against. The current work is part of a scientific investigation on properties of *S*. *platensis* against agricultural crop pests to identify some chemical alternatives for pest control. *Spirulina platensis* is the richest nutrient-containing food since it contains >100 nutrients and holds 62% protein. Such high nutrient values are not present in any type of plant, fruit, or the grains. Due to the reason, it is being heavily used in child’s food [11]. The availability of excessive nutrients might be the reason of shortened larval span and a greater number of eggs in some treatments particularly with higher formulations of *S*. *platensis*.

It has a very beneficial impact on vertebrates like chickens, fish, and even humans. However, its toxic effects on insects also have been reported. *S*. *platensis* caused malformation of the larvae and pupae in cotton leafworm, *Spodoptera littoralis* (Boisd.) with 5% formulation resulting in 100% larval mortality [19]. Similarly, when green tiger shrimp were fed on *S*. *platensis* powder mix diet, 44% of them died [13].

## Conclusion

*Spirulina platensis* has a favorable influence on the growth of almond moth larvae fed at lower amount. However, 5% or higher formulation in diet caused significant mortality. Furthermore, *Spirulina* as a nutritional supplement posed beneficial impacts on life history traits of surviving larvae. More research is needed to investigate the harmful effects of *S*. *platensis* at higher formulation on all developmental stages of almond moth.
